# Influence of DNA methylation on positioning and DNA flexibility of nucleosomes with pericentric satellite DNA

**DOI:** 10.1098/rsob.150128

**Published:** 2015-10-07

**Authors:** Akihisa Osakabe, Fumiya Adachi, Yasuhiro Arimura, Kazumitsu Maehara, Yasuyuki Ohkawa, Hitoshi Kurumizaka

**Affiliations:** 1 Laboratory of Structural Biology, Graduate School of Advanced Science and Engineering, Waseda University, 2-2 Wakamatsu-cho, Shinjuku-ku, Tokyo 162-8480, Japan; 2 Department of Advanced Medical Initiatives, Faculty of Medicine, Kyushu University, Fukuoka 812-8582, Japan

**Keywords:** chromatin, nucleosome, pericentric heterochromatin, DNA methylation

## Abstract

DNA methylation occurs on CpG sites and is important to form pericentric heterochromatin domains. The satellite 2 sequence, containing seven CpG sites, is located in the pericentric region of human chromosome 1 and is highly methylated in normal cells. In contrast, the satellite 2 region is reportedly hypomethylated in cancer cells, suggesting that the methylation status may affect the chromatin structure around the pericentric regions in tumours. In this study, we mapped the nucleosome positioning on the satellite 2 sequence *in vitro* and found that DNA methylation modestly affects the distribution of the nucleosome positioning. The micrococcal nuclease assay revealed that the DNA end flexibility of the nucleosomes changes, depending on the DNA methylation status. However, the structures and thermal stabilities of the nucleosomes are unaffected by DNA methylation. These findings provide new information to understand how DNA methylation functions in regulating pericentric heterochromatin formation and maintenance in normal and malignant cells.

## 1. Introduction

DNA methylation is an important epigenetic mark that regulates the formation of chromatin domains, such as heterochromatin [1–5]. In mammals, DNA methylation occurs in the CpG dinucleotide and is considered to affect the structure and stability of the nucleosome, which is the basic architecture in chromatin [6–10]. In the nucleosome, about 150 base pairs of DNA are left-handedly wrapped around the histone octamer, composed of two each of the core histones H2A, H2B, H3 and H4 [11–13].

DNA methylation is reportedly correlated with nucleosome positioning in plant and mammalian genomes [14,15]. The genomic DNA regions with high CpG content are known as CpG islands, and the CpG methylation apparently plays pivotal roles in gene regulation and genomic DNA maintenance [4,16,17]. Abnormal DNA methylation statuses have been detected in various cancer cells [18,19]. CpG islands are mostly hypomethylated in normal cells, but are hypermethylated in cancer cells, especially in the promoters of tumour suppressor genes [4,20,21]. In contrast, large-scale CpG island demethylation has been detected at the tissue-specific gene promoters in lung cancers [22]. These previous findings suggested that DNA methylation functions in proper gene expression and genomic DNA stability [23,24].

Heterochromatin instability in pericentromeric satellite regions has also been detected as an early and frequent event during human carcinogenesis [25]. Interestingly, this heterochromatin instability occurs concomitantly with the hypomethylation of the CpG sites on the satellite DNA [25–28]. However, the means by which this difference in the CpG methylation status affects the structural features of the nucleosome remain elusive.

In this study, we reconstituted nucleosomes with methylated and unmethylated human satellite 2 DNA fragments, in which the CpG sites are reportedly hypomethylated in cellular carcinomas [29]. Our biochemical and structural analyses revealed that the DNA methylation influenced the positioning and the DNA end flexibility of the nucleosomes assembled on the satellite 2 sequence, without affecting the nucleosome structures and stabilities.

## 2. Results

### 2.1. Nucleosome formation on the human satellite 2 sequence

**Figure 1.**
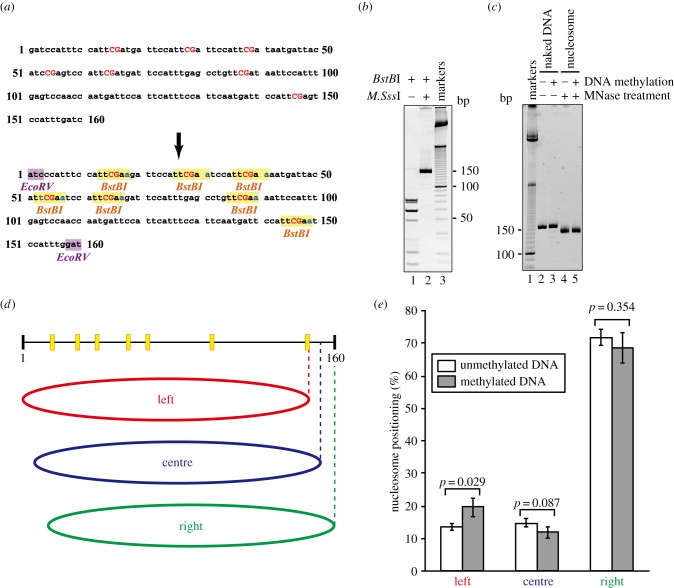
Translational positions of nucleosomes on methylated and unmethylated human satellite 2 DNAs. (*a*) Human pericentric satellite 2 DNA sequence (NCBI accession code: 603562, upper panel). The seven CpG sites are represented by capital red letters. The satellite 2 derivative (Sat2), in which the seven CpG sites of the satellite 2 DNA were substituted with *BstB*I recognition sites (highlighted by yellow rectangles), is represented in the lower panel. For DNA fragment preparation, Sat2 contains *Eco*RV sites at both ends of the DNA (highlighted by purple rectangles). (*b*) Non-denaturing PAGE analysis of the methylated and unmethylated Sat2 DNAs. The DNA fragment was methylated by the *M.Sss*I DNA methyltransferase, and then treated with the *BstB*I restriction enzyme (8 units µg^−1^ DNA, lane 2). Lane 1 indicates a control experiment without *M.Sss*I. The DNA (200 ng) was analysed by 10% PAGE with ethidium bromide staining. Lane 3 indicates the 10 base-pair DNA ladder markers. (*c*) The methylated and unmethyated DNA fragments (30 ng), with or without MNase treatment, were analysed by non-denaturing PAGE. Lane 1 indicates the 10 base-pair DNA ladder markers. Lanes 2 and 3 indicate the unmethylated and methylated Sat2 DNA fragments. Lanes 4 and 5 indicate the nucleosomal unmethylated and methylated Sat2 DNA fragments, protected from MNase. (*d*) Schematic of the translational nucleosome positions, determined by deep sequencing after MNase treatment. Yellow boxes indicate the CpG sites. The red (left), blue (centre) and green (right) ellipses represent the three translational nucleosome positions, with dyad axes located in the 75 (±3), 81 (±3) and 88 (±3) regions, respectively. (*e*) Graphic representation of the nucleosome ratios, located at the left, centre and right positions. White and grey bars represent the experiments with unmethylated and methylated Sat2 DNAs, respectively. Standard deviation values are shown (*n* = 3).

We first prepared a 160 base-pair human satellite 2 DNA fragment. This satellite 2 fragment contained seven CpG sites, TTCGAT, TTCGAT, TTCGAT, TCCGAG, TTCGAT, TTCGAT and TTCGAG (from 5′ to 3′), which are potentially methylated in normal cells (figure 1*a*, upper panel). To ensure that these CpG sites are fully methylated, all of the CpG sites were replaced by TTCGAA, which can be cleaved by the restriction enzyme *BstB*I (figure 1*a*, lower panel). In this study, this satellite 2 derivative was named Sat2. As shown in figure 1*b* (lane 1), all of the CpG sites in the Sat2 160 base-pair fragment were digested by *BstB*I. As anticipated, the *BstB*I cleavage was completely inhibited when the Sat2 160 base-pair fragment was treated with the DNA methyltransferase *M.Sss*I (figure 1*b*, lane 2), indicating that all seven CpG sites of Sat2 were fully methylated.

We then reconstituted the nucleosomes with methylated or unmethylated 160 base-pair Sat2 DNA fragments, by the salt dialysis method. The reconstituted nucleosomes were treated with micrococcal nuclease (MNase), which preferentially cleaves the linker DNA segments detached from the histone surface, and the resulting approximately 145 base-pair DNA fragments were purified (figure 1*c*, lanes 4 and 5). We then performed massively parallel sequencing (deep sequencing) with these MNase-treated DNA fragments and found one major (right, denoted as R) and two minor (centre and left, denoted as C and L, respectively) nucleosome positions on the Sat2 sequence (figure 1*d*,*e*). The major R position was mapped on the right edge of the Sat2 DNA fragment, and the minor C and L positions were shifted by about 7 and 13 base pairs from the right edge, respectively (figure 1*d*). In both the methylated and unmethylated Sat2 DNAs, about 70% of the nucleosomes were formed at the R position, although a slight decrease was observed with the methylated Sat2 (figure 1*e*). Similarly, upon the DNA methylation, the nucleosome population at the C position was decreased (figure 1*e*). In contrast, the population of the L position was increased 1.5-fold when the methylated Sat2 was used as the substrate (figure 1*e*).

### 2.2. Crystal structures of the nucleosomes containing the methylated Sat2R and Sat2L DNAs

**Figure 2.**
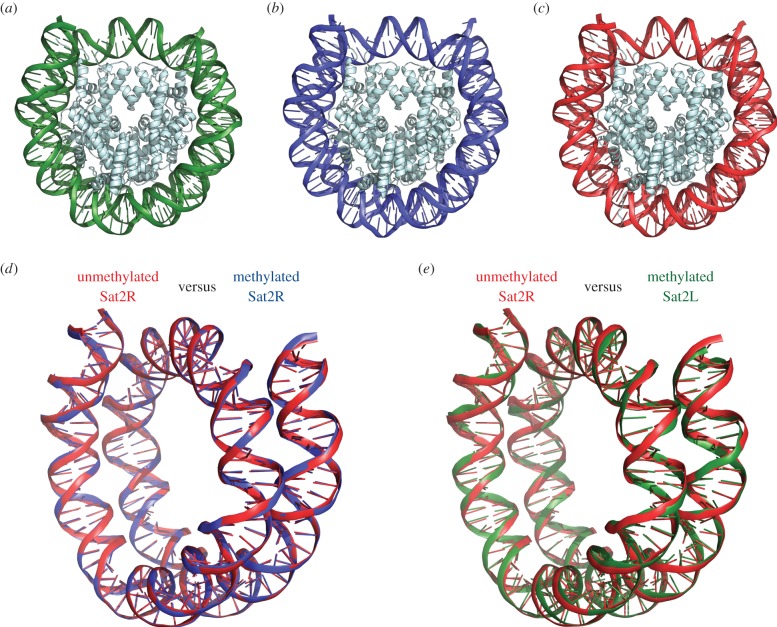
Crystal structures of nucleosomes containing methylated Sat2 DNAs. (*a,b*) The crystal structures of the nucleosomes containing the methylated satellite 2 left (Sat2L) DNA (*a*) and the methylated satellite 2 right (Sat2R) DNA (*b*). The 5-methyl-cytosines were not visible in these structures, because the nucleosomes were packed in a nested manner in the crystals. (*c*) The crystal structure of the nucleosome containing unmethylated Sat2R DNA. (*d*) The methylated Sat2R DNA structure is superimposed on the unmethylated Sat2R DNA structure in the nucleosomes. (*e*) The methylated Sat2L DNA structure is superimposed on the unmethylated Sat2R DNA structure in the nucleosomes.

We crystallized the nucleosomes containing the methylated Sat2L (145 base pairs) and Sat2R (146 base pairs) DNA fragments and determined their structures at 2.63 Å and 3.15 Å resolutions, respectively (table 1 and figure 2*a*,*b*). For a reference, we also determined the structure of the nucleosome containing the unmethylated Sat2R sequence at 2.90 Å resolution (table 1 and figure 2*c*). The histone octamer structures in the nucleosomes containing the methylated Sat2R and Sat2L DNAs were the same as that in the nucleosome containing the unmethylated Sat2R DNA (figure 2*a*–*c*). In addition, the DNA binding path in the methylated Sat2R nucleosome was not different from that in the unmethylated R nucleosome (figure 2*d*). The DNA binding path in the methylated Sat2L nucleosome was also the same as that in the unmethylated Sat2R nucleosome (figure 2*e*). Therefore, these results indicate that the hypermethylation at the seven CpG positions of the Sat2 DNA does not affect the intrinsic DNA wrapping property of the histone octamer.

**Table 1.** Data collection and refinement statistics (molecular replacement).

	unmethylated Sat2R nucleosome	methylated Sat2R nucleosome	methylated Sat2L nucleosome
resolution range (Å)	50–2.90	50–3.15	50–2.63
space group	P2_1_2_1_2_1_	P2_1_2_1_2_1_	P2_1_2_1_2_1_
cell parameters	*a* = 105.431 Å; *b* = 109.331 Å; *c* = 175.771 Å; *α* = 90.0°; *β* = 90.0°, *γ* = 90.0°	*a* = 103.452 Å; *b* = 108.990 Å; *c* = 173.446 Å; *α* = 90.0°; *β* = 90.0°, *γ* = 90.0°	*a* = 105.197 Å; *b* = 109.297 Å; *c* = 173.686 Å; *α* = 90.0°; *β* = 90.0°, *γ* = 90.0°
total number of unique reflections	44 980	34 975	59 234
*R*_merge_ (%)^a^	9.0 (48.6)	8.7 (48.3)	7.1 (48.9)
completeness (%)	98.7 (97.5)	99.3 (98.3)	98.8 (97.7)
*I*/*σ* (*I*)	12.6 (2.2)	11.6 (2.4)	14.1 (2.3)
redundancy	5.0 (3.5)	5.9 (4.2)	5.3 (3.5)
refinement			
resolution (Å)	37.9–2.90	24.9–3.15	19.9–2.63
*R*_work_/*R*_free_ (%)^b^	25.12/29.49	23.52/29.55	22.38/28.42
r.m.s.d. bonds (Å)	0.010	0.010	0.010
r.m.s.d. angles (°)	1.158	1.331	1.369
Ramachandran plot			
most favoured (%)	97.96	96.58	97.95
allowed (%)	2.04	3.42	2.05
disallowed (%)	0	0	0
PDB code	5CPI	5CPJ	5CPK

^a^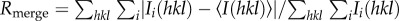.

^b^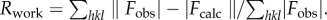 *R*_free_ was calculated with 5% of the data excluded from the refinement.

**Figure 3.**
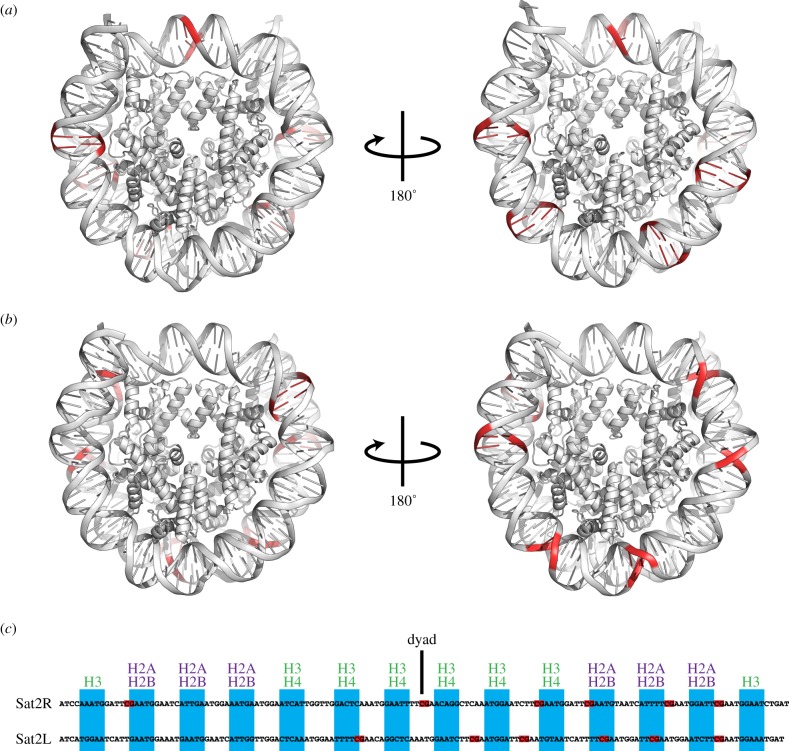
Locations of 5-methyl-cytosines in the Sat2R and Sat2L nucleosomes. (*a*,*b*) Two views of the Sat2R nucleosome (*a*) and the Sat2L nucleosome (*b*). The CpG sites of the nucleosomal DNAs are coloured red. (*c*) Alignment of the Sat2R and Sat2L DNAs. The CpG sites are coloured red. According to the method reported by Chua *et al.* [30], the 5 base-pair segments that directly contact the histone surface are represented by blue rectangles. Histone species contacting the DNA segments are represented at the top of the panel.

Since the nucleosomes were packed in a nested manner in the crystals, the additional methyl groups of the 5-methyl-cytosines were not visible in these nucleosome structures. Therefore, we mapped the 5-methyl-cytosine locations on these nucleosome structures in two nested orientations (figure 3*a*,*b*). Six out of the seven CpG sites were incorporated into each SatR or SatL nucleosome (figure 3*c*). Interestingly, in the Sat2R nucleosome, most of the 5-methyl-cytosines tended to be exposed to the solvent (figure *3c*). In contrast, two 5-methyl-cytosines are buried in the histone–DNA contact surface in the Sat2L nucleosome (figure 3*c*). These structural differences may affect the accessibility of the methyl-DNA binding proteins to the nucleosomal 5-methyl-cytosine [31].

### 2.3. DNA methylation changes the accessibility of the DNA ends of the nucleosome, without affecting its thermal stability

**Figure 4.**
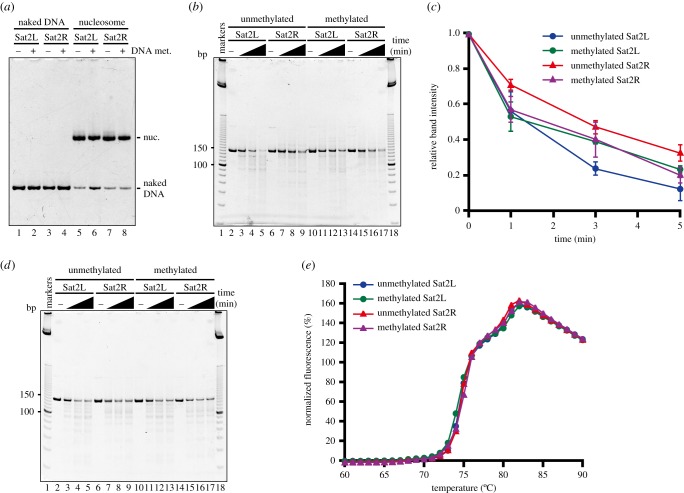
The MNase assay. (*a*) Purified nucleosomes containing unmethylated Sat2L DNA (lane 5), methylated Sat2L DNA (lane 6), unmethylated Sat2R DNA (lane 7) and methylated Sat2R DNA (lane 8) were analysed by 6% non-denaturing PAGE with ethidium bromide staining. Lanes 1–4 indicate the naked DNAs of unmethylated Sat2L DNA (lane 1), methylated Sat2L DNA (lane 2), unmethylated Sat2R DNA (lane 3) and methylated Sat2R DNA (lane 4), respectively. (*b*) The MNase susceptibility assay with nucleosomes. Purified nucleosomes (200 ng DNA) containing unmethylated Sat2L DNA (lanes 2–5), unmethylated Sat2R DNA (lanes 6–9), methylated Sat2L DNA (lanes 10–13) and methylated Sat2R DNA (lanes 14–17) were treated with MNase (0.8 units) for 0 (lanes 2, 6, 10 and 14), 1 (lanes 3, 7, 11 and 15), 3 (lanes 4, 8, 12 and 16) and 5 min (lanes 5, 9, 13 and 17). Lanes 1 and 18 indicate the 10 base-pair DNA ladder markers. (*c*) Graphic representation of the experiments shown in panel (*b*). Standard deviation values are shown (*n* = 3). (*d*) The MNase susceptibility assay with naked DNAs. Unmethylated Sat2L DNA (lanes 2–5), unmethylated Sat2R DNA (lanes 6–9), methylated Sat2L DNA (lanes 10–13) and methylated Sat2R DNA (lanes 14–17) were treated with MNase (0.04 units) for 0 (lanes 2, 6, 10 and 14), 1 (lanes 3, 7, 11 and 15), 3 (lanes 4, 8, 12 and 16) and 5 min (lanes 5, 9, 13 and 17). Lanes 1 and 18 indicate the 10 base-pair DNA ladder markers. (*e*) Thermal stability curves of the nucleosomes. The normalized fluorescence intensity was plotted against the temperature (from 60°C to 90°C).

We next tested the MNase sensitivity of the nucleosomes containing the methylated and unmethylated DNAs with two R and L positions. To do so, four types of nucleosomes, containing methylated Sat2R (146 base pairs), unmethylated Sat2R (146 base pairs), methylated Sat2L (145 base pairs) and unmethylated Sat2L (145 base pairs) DNAs, were reconstituted and purified by native polyacrylamide gel electrophoresis (PAGE) (figure 4*a*).

The quantitative MNase assay revealed that, under the unmethylated conditions, the nucleosome containing the Sat2L DNA was quite susceptible to MNase, as compared to the nucleosome containing the Sat2R DNA (figure 4*b*,*c*). DNA methylation drastically reduced the MNase susceptibility of the Sat2L nucleosome (figure 4*b*,*c*). In contrast, the MNase susceptibility of the Sat2R nucleosome was enhanced upon DNA methylation (figure 4*b*,*c*). In nucleosomes, MNase is known to preferentially degrade the DNA segments that are detached from the histone surface. We confirmed that MNase equally degraded the non-nucleosomal Sat2L and Sat2R DNAs (figure 4*d*), indicating that the enzyme did not exhibit any sequence specificity to these DNAs. In addition, the DNA methylation did not affect the MNase susceptibility of the non-nucleosomal Sat2L and Sat2R DNAs (figure 4*d*). Therefore, these data indicate that DNA hypermethylation enhances the DNA end flexibility of the Sat2R nucleosome, but reduces that of the Sat2L nucleosome.

Since the thermal stabilities of these four nucleosomes were exactly the same, the differences in their MNase susceptibilities were not due to changes in the nucleosome stability upon DNA methylation (figure 4*e*). In this thermal stability assay, nucleosome disruption by heating was monitored as histone dissociation from the nucleosome, by using SYPRO Orange, a fluorescent dye that specifically binds to denatured proteins, as a probe. Therefore, DNA hypermethylation influenced the DNA end flexibility of the nucleosomes without affecting their thermal stabilities, and this may depend on the translational positioning of the nucleosomes.

## 3. Discussion

The human satellite 2 repeats located in pericentric heterochromatin regions are highly methylated in normal cells, but are reportedly hypomethylated in cancer cells [32–34]. However, the question remained as to whether the DNA methylation status affects the structure and stability of the nucleosome on the satellite 2 sequence. To answer this question, we reconstituted the satellite 2 nucleosomes with or without DNA methylation and studied the impacts of the DNA methylation on the positioning, structure, stability and DNA end flexibility of the nucleosomes.

We identified the major and minor nucleosome positions on the satellite 2 sequence (figure 1*d*,*e*). We found that the nucleosome population of the minor position (L) significantly increased upon DNA methylation (figure 1*d*,*e*). It is intriguing that the Sat2L nucleosome was more easily degraded by MNase in the absence of DNA methylation, without affecting the nucleosome structure and stability (figures 2–4). Therefore, the methylation of the satellite 2 DNA may function to accommodate the DNA ends of the Sat2L nucleosome more tightly. This is consistent with a previous report that DNA methylation reportedly facilitates the wrapping of DNA ends [6,8,35].

However, the DNA methylation oppositely affected the DNA end flexibility of the major Sat2R nucleosome. This is consistent with the results reported by Jimenez-Useche & Yuan [7], who found that the DNA methylation does not compact the nucleosomal DNA [7]. Therefore, the previous controversial observations regarding whether the DNA methylation reduces or enhances the nucleosomal DNA end flexibility may be reconciled, by considering the translational positions of the nucleosome. Consistent with this idea, DNA methylation reportedly affects the DNA end flexibility differently, depending on the nucleosomal locations of the CpG dinucleotides [9].

We found that the DNA end flexibilities of the Sat2R and Sat2L nucleosomes became similar when the satellite 2 DNA was fully methylated. This finding suggests that DNA methylation may reduce the differences in the nucleosome characteristics and may function to facilitate well-organized, regular chromatin folding in heterochromatin.

In this study, we determined the physical characteristics of satellite 2 nucleosomes with or without DNA methylation. Our results have led to a new question: how are these structural and physical characteristics of the satellite 2 nucleosomes with or without DNA methylation linked to the chromosome instability frequently observed in cancer cells? Further cell-biological and genetic studies are awaited.

## 4. Material and methods

### 4.1. Purification of recombinant human histones

Human histones H2A, H2B, H3 and H4 were purified by the method described previously [36–39]. In this method, bacterially expressed human histones with an N-terminal His_6_-tag were purified with Ni-NTA agarose (Qiagen). After removal of the His_6_-tag portion by the addition of thrombin protease (1 unit mg^−1^ protein), the histones were further purified by MonoS column chromatography (GE Healthcare), freeze-dried and stored at 4°C.

The freeze-dried histones (1 : 1 : 1 : 1 stoichiometry) were dissolved in 20 mM Tris–HCl buffer (pH 7.5), containing 7 M guanidine hydrochloride and 20 mM 2-mercaptoethanol. The sample was dialysed against 10 mM Tris–HCl buffer (pH 7.5), containing 2 M NaCl, 1 mM EDTA and 5 mM 2-mercaptoethanol, and the resulting histone octamers were purified by Superdex200 (GE Healthcare) gel filtration column chromatography.

### 4.2. Preparation of Sat2 DNA fragments for nucleosome reconstitution

Four 160 base-pair Sat2 DNA fragments, each bearing seven *BstB*I (New England BioLabs) recognition sites, were inserted into the pGEM-T Easy vector (Promega). The plasmid was amplified in *Escherichia coli* cells and was purified by the method described previously [40]. The 160 base-pair Sat2 DNA fragment was isolated from the plasmid by digestion with *EcoR*V. The vector DNA portion was removed by PEG-6000 precipitation, and the 160 base-pair Sat2 DNA fragment was then purified by chromatography on TSKgel DEAE-5PW (TOSOH). For the Sat2L and Sat2R DNA fragments, eight Sat2L (145 base pairs) or Sat2R (146 base pairs) DNA fragments were tandemly ligated into the pGEM-T Easy vector. The DNA fragments were purified by the same methods as described above.

The DNA sequences of Sat2L and Sat2R were as follows.

Sat2L: 5′–ATCAT TTCCA TTCGA AGATT CCATT CGAAT CCATT CGAAA ATGAT TACAT TCGAA TCCAT TCGAA GATTC CATTT GAGCC TGTTC GAAAA TTCCA TTTGA GTCCA ACCAA TGATT CCATT CATTT CCATT CAATG ATTCC ATGAT–3′.

Sat2R: 5′–ATCAG ATTCC ATTCG AATCC ATTCG AAAAT GATTA CATTC GAATC CATTC GAAGA TTCCA TTTGA GCCTG TTCGA AAATT CCATT TGAGT CCAAC CAATG ATTCC ATTCA TTTCC ATTCA ATGAT TCCAT TCGAA TCCAT TTGGA T–3′.

CpG methylation was introduced by an incubation with the bacterial DNA methyltransferase *M.Sss*I (New England BioLabs), in the presence of 160 µM *S*-adenosylmethionine (2 units µg^−1^ DNA) at 37°C for 16 h. The reaction was terminated by an incubation at 65°C for 30 min. The unmethylated satellite 2 DNA was cleaved with *BstB*I (10 units µg^−1^ DNA) at 65°C for 4 h, and the resulting methylated Sat2 DNA was purified by chromatography on TSKgel DEAE-5PW.

### 4.3. Reconstitution of nucleosomes containing Sat2 DNA fragments

Nucleosomes containing the Sat2 DNA fragments were reconstituted by the salt dialysis method, as described previously [37–39]. DNA fragments were mixed with histone octamers in the presence of 2 M KCl. The KCl concentration was gradually reduced from 2 M to 0.25 M, using a peristaltic pump. The reconstituted nucleosomes were incubated at 55°C for 2 h and were further purified by non-denaturing PAGE, using a Prep Cell apparatus (Bio-Rad).

### 4.4. Deep sequencing analysis of the nucleosome positioning

Purified nucleosomes, containing the unmethylated or methylated Sat2 DNA fragment, were treated with MNase (3 units µg^−1^ DNA). DNA fragments containing about 145 base pairs were extracted and purified by electroelution. The library was prepared using an NEBNext Ultra DNA Library Prep Kit and was then sequenced using an Illumina HiSeq 1500 system (Illumina K.K.; USA). The sequenced reads were uniquely mapped onto the target DNA sequence, using the Bowtie 2 program (v. 2.2.2) with default parameters. The proportions of the mapped reads of the nucleosome dyad position, which was estimated as the position shifted by 73 base pairs from the 5′-end of the reads, on the target Sat2 DNA (1–160 base pairs), were calculated.

### 4.5. Thermal stability assay for nucleosomes

The nucleosome stability was monitored by a thermal stability assay, as described previously [41–43]. Purified nucleosomes (1.1 µM) were mixed with SYPRO Orange dye (Sigma-Aldrich) in 20 mM Tris–HCl buffer (pH 7.5), containing 1 mM DTT. The SYPRO Orange fluorescence was detected with a StepOnePlus™ Real-Time PCR unit (Applied Biosystems), using a temperature gradient from 25°C to 95°C, in steps of 1°C min^−1^.

### 4.6. Crystallization and structure determination

Purified nucleosomes containing unmethylated or methylated Sat2R DNA (146 base pairs) and methylated Sat2L DNA (145 base pairs) fragments were dialysed against 20 mM potassium cacodylate buffer (pH 6.0), containing 1 mM EDTA. The nucleosome solution (3.5 mg ml^−1^ DNA concentration) was mixed with an equal volume of 20 mM potassium cacodylate buffer (pH 6.0), containing 50–70 mM KCl and 70–105 mM MnCl_2_. The drops were equilibrated against 500 µl of reservoir solution (20 mM potassium cacodylate buffer (pH 6.0), containing 35–45 mM KCl and 45–60 mM MnCl_2_), and crystals were obtained by the hanging drop method. The resulting nucleosome crystals were cryoprotected by soaking in a solution containing 20 mM potassium cacodylate (pH 6.0), 35–40 mM KCl, 50–60 mM MnCl_2_, 28% (+/−)-2-methyl-2,4-pentanediol and 2% trehalose, and were flash-cooled in a stream of N_2_ gas (100 K). The diffraction data of the nucleosomes containing the unmethylated Sat2R DNA and the methylated Sat2L DNA were collected on the BL17A (wavelength: 0.98000 Å) at the Photon Factory (Tsukuba, Japan). The diffraction datasets of the nucleosome containing the methylated Sat2R DNA fragment were collected on the BL41XU (wavelength: 1.00000 Å) at SPring-8 (Harima, Japan). The datasets were processed using the HKL2000 and CCP4 programs [44,45]. The structures of the nucleosomes containing the methylated and unmethylated 146 base-pair Sat2R DNA fragments were determined by molecular replacement with the PHASER program, using the crystal structure of the nucleosome containing the 146 base-pair DNA (PDB ID: 3AFA) as the search model [37,46]. In the case of the nucleosome containing the methylated 145 base-pair Sat2L DNA fragment, the crystal structure of the nucleosome containing the 145 base-pair DNA (PDB ID: 3UT9) was used as the search model for molecular replacement [30]. The refinements of the atomic coordinates were performed using the PHENIX, CNS and Coot programs [47–49]. Structural graphics were displayed using the PyMOL program (http://pymol.org). The atomic coordinates of the unmethylated Sat2R nucleosome, the methylated Sat2R nucleosome and the methylated Sat2L nucleosome have been deposited in the Protein Data Bank, with the ID codes 5CPI, 5CPJ and 5CPK, respectively.

### 4.7. Micrococcal nuclease treatment assays

The nucleosomes containing unmethylated Sat2R, unmethylated Sat2L, methylated Sat2R and methylated Sat2L DNA (200 ng DNA) were incubated with MNase (0.8 units) in 10 µl of 50 mM Tris–HCl (pH 8.0) buffer, containing 2.5 mM CaCl_2_ and 0.9 mM dithiothreitol, at 25°C for 1, 3 and 5 min. For the experiments with naked DNAs, unmethylated Sat2R, unmethylated Sat2L, methylated Sat2R and methylated Sat2L DNA (200 ng DNA) were incubated with MNase (0.04 units) in 10 µl of 50 mM Tris–HCl (pH 8.0) buffer, containing 2.5 mM CaCl_2_ and 0.9 mM dithiothreitol, at 25°C for 1, 3 and 5 min. After the incubation, the reactions were stopped by the addition of stop solution (60 µl), composed of 20 mM Tris–HCl (pH 8.0), 20 mM EDTA, 0.25% SDS and 0.5 mg ml^−1^ proteinase K (Roche). The reaction mixtures were further incubated at 25°C for 15 min. The DNA was then extracted with phenol–chloroform, and the resulting DNA fragments were analysed by 10% non-denaturing PAGE in 0.5× TBE buffer (45 mM Tris base, 45 mM boric acid and 1 mM EDTA). The DNA bands were visualized by ethidium bromide staining.

## Data accessibility

PDB IDs: The atomic coordinates of the unmethylated Sat2R, methylated Sat2R and methylated Sat2L nucleosomes have been deposited in the Protein Data Bank, with the ID codes 5CPI, 5CPJ and 5CPK, respectively.
